# An unexpected complication after removing bladder foreign body: a case report

**DOI:** 10.3389/fsurg.2024.1405129

**Published:** 2024-08-02

**Authors:** Pengfeng Gong, Jie Shen

**Affiliations:** Department of Urology, The Third Affiliated Hospital of Soochow University, Changzhou, Jiangsu, China

**Keywords:** bladder, foreign bodies, serious complications, cystoscopy, laparoscopy

## Abstract

**Background:**

Bladder foreign bodies commonly arise as urgent issues in urology. These foreign bodies are typically extracted through cystoscopy or cystotomy. In general, these surgical approaches rarely lead to serious complications.

**Methods:**

A 34-year-old woman presented with a one-year history of frequent urination, urgency, and urodynia. Abdominal computed tomography (CT) scan revealed the presence of an intrauterine device (IUD) [a medium-sized (20 mm × 22 mm) circular IUD] near the posterior bladder wall. The object was successfully removed via cystoscopy. Two months later, the patient exhibited food residues in her urine. Enterography demonstrated a large amount of contrast agent had entered the bladder from the small intestine. We repaired the bladder with catheter for 2 weeks, removed the segment of small intestine with fistula, and anastomosed the intestine canal.

**Results:**

Post-operation urine tests yielded negative results, and the patient resumed a normal diet.

**Conclusions:**

Evaluating the location between foreign body and bladder wall, which is based on medical history, CT scan, and cystoscopy examination, is essential for doctors before they remove the foreign body by cystoscopy or laparoscopy. It is necessary to check for leakage by applying radiopaque fluids under fluoroscopy after removing the foreign body, which migrates from other abdominal organs. If there is damage in the bladder or other organs, laparoscopic surgery or open surgery should be performed immediately.

## Introduction

Bladder foreign bodies are sporadic in urology, stemming from diverse causes such as self-insertion, iatrogenic factors, migration from adjacent areas, and assault (1). In the category of foreign bodies migrating to the bladder, intrauterine devices (IUDs) are prevalent. Typically, these are safely extracted via cystoscopy with minimal complications. However, a female patient in our department developed a vesicocenteric fistula following the removal of a foreign body via cystoscopy.

## Case series presentation

### Description of patient

A 34-year-old woman experienced frequent urination, urgency, and urodynia for a year, and an IUD was placed in her uterus directly for contraception in outpatient 3 years ago. But she did not undergo a follow-up ultrasound at the hospital one month after the placement of IUD to confirm that it was in the uterus.

### Case history

After antibiotic treatment, the patient’s urinary tract infection kept recurring, so she received the abdominal computed tomography (CT) scan in another hospital. The abdominal CT revealed the presence of an IUD and unclear boundaries between the IUD and the posterior wall of the bladder.

### Diagnostic assessments

The patient came to our department for hospitalization. After anti-infection treatment for bladder, we performed a cystoscopy on her. We found an IUD embedded in the bladder mucosa and muscle layer, and approximately one-third of the volume of the IUD was inside the bladder.

### Therapeutic interventions

We removed the IUD by foreign body forceps through the cystoscopy. After the removal, the patient did not have frequent urination, urgency, urodynia, fever, or any other uncomfortable syndromes.

### Outcome

**Figure 1:**
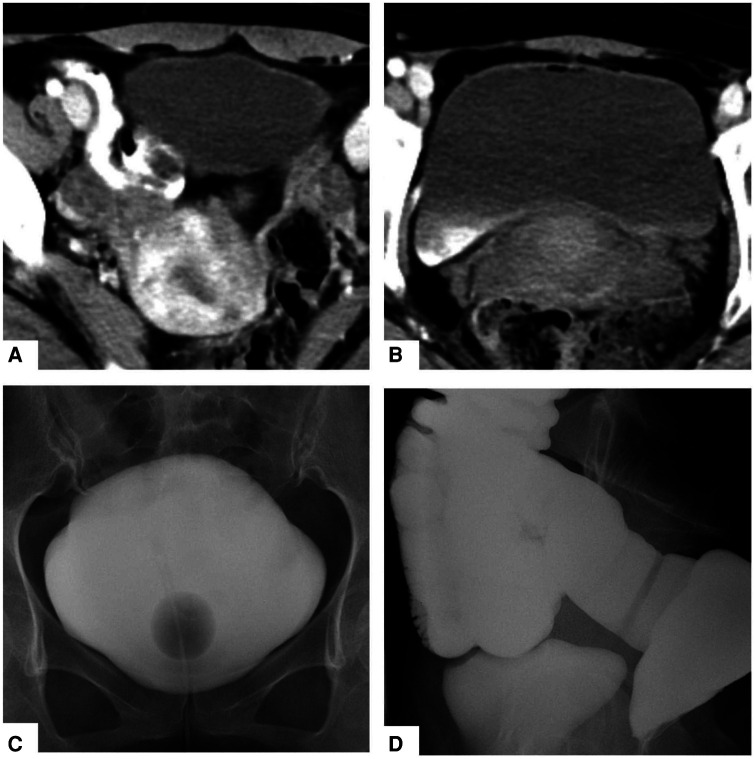
Abdominal CT revealed that a small amount of contrast agent had drained into the bladder from the small intestine, and there was a small amount of air in the bladder (**A**,**B**). Cystography showed that no contrast medium entered the small intestine from the bladder (**C**), but enterography clearly showed that a large amount of contrast medium had entered the bladder from the small intestine (**D**).

Two months after IUD removal, the patient presented with food residues in her urine, leading to her readmission to our department. Cystography indicated no contrast agent entering the intestine from the bladder. However, abdominal CT scan revealed a small amount of contrast agent migrating into the bladder from the small intestine, accompanied by a small amount of air in the bladder (Figure 1). Enterography displayed a substantial amount of contrast agent entering the bladder from the small intestine (Figure 1). Considering the patient’s medical history, clinical signs, and imaging findings, a diagnosis of bladder–small bowel fistula was established. We decided to perform open surgery. According to CT images, the bladder–small intestine fistula was located at the junction of the posterior and right walls of the bladder in the abdominal cavity. The incision was located in the middle of the lower abdomen. After opening the abdominal cavity, we found obvious adhesions between the bladder, uterus, and small intestine. We carefully released these adhesions and discovered the bladder–small bowel fistula in the junction of the right and posterior walls of the bladder. After separating the small intestine and bladder, we repaired the bladder with absorbable thread with catheter for 2 weeks. We removed the segment of small intestine with fistula, which cannot be repaired, and anastomosed the intestine canal. The patient experienced a satisfactory recovery and was discharged from the hospital 10 days after the operation.

### Description about follow-up

After 1 year of post-operative follow-up, the patient did not present any further symptoms such as frequent urination, urgency, and urodynia, and there were no food residues in her urine.

## Discussion

The migration of foreign bodies from other organs to the bladder stands as a prevalent cause of bladder foreign bodies. A study from India documented four cases of IUD migrating from the uterus to the bladder, all of which were successfully managed through cystoscopy. In our prior encounters with seven female patients having an IUD in the bladder, treated solely with cystoscopy and without laparoscopy, no serious complications were observed. Based on past experiences, we assumed that IUD can be easily removed through cystoscopy. Although an IUD embedded in the bladder mucosa and muscle layer and its removal may cause damage to the bladder wall, we believe that the bladder injury will cure by itself with the use of catheter for 2 weeks. It was completely beyond our expectations when the bladder–small intestine fistula formed 2 months after removal, which we have never encountered before. In the previous reports, there are some treatments, which is different from that of our case. A study from South Korea underscored the utility of laparoscopy in cases involving foreign body migration to the bladder. This approach allows for a clear assessment of bowel perforation or fistula, precise localization, and visualization of the relationship among foreign bodies, the bladder wall, and adjacent organs. Consequently, clinicians can make informed decisions regarding laparoscopic repair of the bladder, intestines, or other affected organs (2). Jin et al. (3) presented a case wherein foreign bodies embedded in the urinary bladder wall were successfully extracted using a combination of laparoscopy and carbon dioxide cystoscopic assistance. As reported by Lin et al. (4), they performed a laparoscopy for retrieval of IUD, because IUD was mainly located in the abdominal cavity, with only a small portion embedded in the bladder wall, and prepared for a possible concurrent urologic procedure.

Yennie et al. (5) performed cystoscopy and found that the long limb of the IUD was embedded in the mucosal and muscular layers, such that it could not be removal by cystoscopy.

In summary, the imperative step prior to removal of foreign body from a bladder, migrating from other abdominal organs, is an abdominal CT and cystoscopy, elucidating the positional relationship between the foreign body and the bladder, along with surrounding organs. In addition, it is necessary for doctors to enquire about the medical history in detail, including its size, shape, type, and so on, which may decide the treatment. As presented by Lin et al. the shape and location of IUD determined that it cannot be removal by cystoscopy (4). Laparoscopic exploration is a good choice when the foreign body cannot be removed by cystoscopy, while preparing for a urologic procedure at the same time. If the foreign body is removed by cystoscopy, it is appropriate to check for leakage or fistula by applying radiopaque fluids under fluoroscopy. And performing a pre-operative contrast-enhanced magnetic resonance imaging (MRI) is beneficial to seeing the location of the enterovesical fistula and the anatomical structures more clearly so that the surgery can be performed more accurately.

## Conclusion

Concerning foreign bodies migrating from other abdominal organs to the bladder, cystoscopy or laparoscopy is generally used to remove them. Evaluate the relationship between bladder foreign body and bladder wall based on medical history, CT scan, and cystoscopy examination, and choose the appropriate removal method. After the removal of the foreign body, do not forget to check for leakage or fistula by applying radiopaque fluids under fluoroscopy. If damage of the bladder or other organs is found, the patient should undergo open surgery or laparoscopic surgery immediately.

## Data Availability

The original contributions presented in the study are included in the article/Supplementary Material, further inquiries can be directed to the corresponding author.
